# An interview with Professor Benjamin Becker: understanding our brain and mental disorders requires collaboration across all disciplines

**DOI:** 10.1093/psyrad/kkae023

**Published:** 2024-11-04

**Authors:** Long-Biao Cui (崔龙彪)

**Affiliations:** Research Fellow at Shaanxi Provincial Key Laboratory of Clinical Genetics, Fourth Military Medical University, Xi'an 710032, China; Research Fellow at Schizophrenia Imaging Laboratory, Xijing 986 Hospital, Fourth Military Medical University 710054, Xi'an, China

## Abstract

From July 20 to 22, 2024, the ISMRM Endorsed Workshop on MR for Psychiatry was held in Chengdu City, China. This prestigious event attracted numerous academic elites worldwide, and Professor Benjamin Becker from the University of Hong Kong was invited. On the morning of July 20, during the “Advances in MR Technology” session, Professor Becker delivered an engaging lecture entitled “Novel approaches to precision MRI-imaging of human emotion.” His presentation was met with great enthusiasm and sparked lively discussions among the participants. Following the conference, the *Psychoradiology* journal interviewed Professor Becker. In the interview, Benjamin emphasized the significant role of interdisciplinary collaboration, spanning various fields including psychology, neuroscience, clinical medicine, biomedical engineering, and computer science. Professor Becker firmly believed that such collaboration was crucial for a deeper understanding of the brain and psychiatric disorders. Additionally, he highly valued the importance of international cooperation, especially in addressing global mental health issues and challenges related to psychiatric disorders.

## Exploring the Evolution of MRI Technology

**1. *Psychoradiology*: How do you enter your research field? What led you to develop an interest in this area?**

**Ben:** How I entered my research field was not relatively simple as one might think. As a student, my primary focus was on personality disorders at that time. I completed my Master's degree, which was totally without imaging. Even then, I became quite interested in the differences between people who are quite good at controlling their emotions and those who are unable control them and are overwhelmed by their emotions. My fascination grew when I participated in an fMRI (functional magnetic resonance imaging) study focused on cognition over 20 years ago. It was during this study that I was struck by the incredible capability of MRI machines to observe brain activity in real-time.

This ability provided a window into how people think and feel, which was particularly captivating because emotions are often seen as elusive and hard to capture compared to cognitive processes, such as those measured by IQ (intelligence quotient) tests. The most basic form of human consciousness is one's emotional experience, because if you feel something, if you are afraid of something, or if you like something, it is always in relation to yourself. I was then amazed by that we can use technology to actually look at the biological basis. Bridging the biological underpinnings with their actual subjective experiences was quite interesting. During my second experience working with patients, I realized that the key reasons for them to seek treatments is often due to strong negative emotion. Therefore, I became interested in what goes wrong in the brain when people can't control their emotions anymore. Emotions, arguably the most fundamental aspect of human consciousness, are deeply intertwined with our biological makeup.

**2. *Psychoradiology*: What is your perspective on the development of this field over the past few decades? What are the significant changes?**

**Ben:** The field of cognitive neuroscience and neuroimaging is a rapidly evolving domain with substantial developments. For instance, a few years ago, the replication crisis was a major topic, with many results in our field being non-replicable at that time. Currently, we are moving away from traditional studies to more network-based measures and machine learning-based measures like MVPA (multi-voxel pattern analysis). We are also moving towards large-scale studies like the UK Biobank and integrating epigenetic factors with brain factors. My fascination with my research field is its critical nature. When faced with challenges like replication issues, the entire field mobilizes to adapt and advance.

**3. *Psychoradiology*: What will be the new challenges and opportunities in this field in the next 5 years?**

**Ben:** There will be numerous challenges, yet many significant ones have been addressed in recent years. The forthcoming challenge for the next 5 years, I believe, lies in integrating MRI into tangible applications, spanning clinical uses to areas such as neurological disorders and brain–computer interfaces. We have invested a huge amount of time and funding into MRI and we are now approaching a stage where we have relatively robust markers. The question now is how to apply these markers to make a difference, particularly for mental disorders.

**4. *Psychoradiology*: Which imaging technologies do you think will have a huge impact on your field of research?**

**Ben:** In terms of imaging, I am now focusing on specific advancements. I believe the development of new MRI systems that provide a better technological platform will have a significant impact. Several large companies and initiatives are working on this, so improving imaging technology is crucial. Additionally, I am very sure that AI (artificial intelligence) will have a huge influence. Complex methods, including machine learning and increasingly advanced AI, are essential for extracting robust markers from the brain, psychiatric disorders, and mental processes like emotions in my field. It makes sense that our work with such complex conscious processes and data requires highly specialized methods to identify clear signatures. For clinical applications, brain stimulation methods are a very important step forward. Such as TMS (transcranial magnetic stimulation), are becoming more relevant and are now approved for several disorders. In the long term, we could potentially have MRI-guided modifications of specific brain regions, possibly through techniques like planting virus to specifically change receptor profiles in specific brain regions. However, developing such techniques will undoubtedly take considerable time.

## Interdisciplinary and International Collaborations Fuel Brain Research Advancements

**5. *Psychoradiology*: What is the role of interdisciplinary collaboration in advancing scientific progress?**

**Ben:** Interdisciplinary collaboration is vital. At our brain imaging conferences, we have professionals from various fields. The brain is an incredibly complex organ, so we need different perspectives on this. For instance, psychological or neuroscience perspective from basic research helps us understand how brain systems work together, how humans feel, how human process emotions, and how we can measure these processes. We need obviously the medical perspective, particularly when it comes to psychiatric disorders of mental dysfunction. We also need biomedical perspective because the brain is fundamentally a biological organ, and we are measuring biological processes. We also need MRI physicists to help find right sequences, engineers to set up the technology, and computer scientists to develop mathematical modeling and computational modeling. This brings together almost 10 different fields, and that is not even all the disciplines involved. Over the past decade, it has been interesting to see how people from entirely different fields come in and suddenly come up with a new perspective that then is captured by the entire field. In summary, understanding the brain and mental disorders requires collaboration across all these disciplines. Even with this interdisciplinary approach, achieving a deep understanding will still be a long process.

**6. *Psychoradiology*: Can you share with us some of your successful experience of interdisciplinary collaboration?**

**Ben:** I think interdisciplinary collaboration is basically key to most of my studies. We have been working very successfully with teams from the USA specializing in machine learning, which has helped us to come up with clear methods to decode the brain. We have also collaborated with Professor Feng Jianfeng's team at Fudan University on large-scale data analysis. This collaboration has been beneficial for our own specific experiments, allowing us to leverage this large-scale data for targeted development. Additionally, our collaborations with medical doctors from psychiatry and mental health hospitals have been productive. We work together to develop ideas, identify pressing mental health issues, and conduct studies with patients, moving on from basic research to applied research. Gathering diverse perspectives is crucial to discern how the brain operates in general terms, rather than focusing solely on its function within specific populations, such as the German or Chinese brain. Engaging in discussions with experts from various countries consistently provides different perspectives, which makes the process truly fascinating.

**7. *Psychoradiology*: In the era of globalization, what is your opinion on the importance of international cooperation in scientific research?**

**Ben:** International cooperation is important in scientific research. Mental health and disorders are a global issue. Over recent years, the numbers have been rising, and mental disorders now become a major global burden of disease. The challenge has grown even more significant after COVID (Coronavirus Disease 2019), with additional factors like climate change and societal changes. At present, we lack highly effective treatments for all patients, a challenge that is recognized worldwide. To address this, we must collaborate, integrating diverse perspectives and working across national boundaries. Our goal is to understand how humans process emotions or how humans think in general, not just those specific countries like Germany or China. Therefore, my team has recently implemented a cross-cultural validation approach comparing brain markers found in different global populations. For example, in the last study on aging-related changes, we analyzed and compared samples from both China and Europe. The presence of convergent brain changes allows us to identify a marker as universally applicable. This methodology is of great significance because a marker that remains consistent across diverse populations, despite differing environmental influences and cultural contexts, can be considered a real biological marker.

**8. *Psychoradiology*: How will Open Access and other publishing models reshape academia in the future?**

**Ben:** Academic publishing is undergoing significant changes in recent years, moving away from traditional models, which is very encouraging. Open Access publishing is an important step forward, eliminating paywalls, making research more accessible. Research aims to solve problems in the end in lots of fields, whether basic or applied. Freely sharing this knowledge is essential for addressing emerging issues. One of the good examples is how the COVID vaccinations were developed. It is based on a basic research procedure that enabled other researchers to quickly develop a response to the COVID. Initially, the practical application of this research was uncertain. Additionally, it is crucial that findings, often funded by public taxes, should be accessible to all, not restricted by paywalls. Two key changes are needed: first, presenting scientific discoveries in an understandable language for the general public; second, ensuring that interested individuals can access these findings without barriers.

## Suggestions to the Young Scientists in the Field

**9. *Psychoradiology*: What are your suggestions for young researchers when they consider their research directions and professional growth?**

**Ben:** I think one of the most important things is to do something that you find interesting and that you think can make a difference. Because if you want to become a good or established researcher in your key fields, it will take you a huge amount of time and you have to work on this topic over years. There are lots of frustrating moments when you are very tired, and when your experiments fail. You might have the moments when you long for a break, but face multiple challenges at the same time. Research is not always smooth sailing; often, experiments don't yield the results you hope for, and the feedback may not be as positive as you expected. You invest your time, and work with different experts together, so it will really take time to make an impact in your research field. The key is to do something that you consider interesting and that you think could make a difference, whether it is out of your own curiosity or a fundamental interest in understanding how the brain possesses emotions, or for other people, developing something new for important disorder. If you are not truly passionate about this, it is probably too long of a stormy road.

**10. *Psychoradiology*: How did you survive and recover from the challenges and setbacks during the difficult periods of your research?**

**Ben:** It is really important to have good friends and collaborators, who sometimes share similar experiences with you. As I have become more senior, I meet and talk with people who are really the best researchers in the world. Most of them tell me that their job is really exciting and amazing, but it is still very demanding and frustrating. This is how the field works. So I believe it is crucial to have a strong network of friends and collaborators with whom you can share experiences, realizing you are not alone in your struggles, as many others in the field face similar challenges. Obviously, family is another essential support system. I am fortunate that my wife understands the ups and downs of my work. Her support is invaluable, especially during those frustrating moments. Furthermore, students play a crucial role as well. I have noticed that when I talk with my students after they have had a disheartening experience, such as an unfair rejection of a paper, I truly empathize with their sense of frustration and injustice. These discussions not only allow me to support them but also place me in a position where I need to explain the situation, which is an enlightening experience in itself. Engaging in these conversations with my students also aids me in refining my own understanding and communication of such setback.

**11. *Psychoradiology*: How has your research impacted the public understanding of science?**

**Ben:** I would discuss the broader impact of my field on public perceptions of mental disorders. The entire field has a significant impact on reshaping mental disorders and mental health. For a long time, there was a belief that mental disorders had no biological basis and were instead attributed to concepts like disruptions in “Yin” and “Yang” or “demonic possession.” Neuroimaging has helped change this perception over the past few decades. Based on animal models and particularly human neuroimaging studies, we have known that cognition and emotion have a biological basis, and mental disorders are linked to brain changes. This understanding is crucial for patients, as it provides reassurance that their conditions are not beyond control but have a biological foundation. Recognizing that a mental disorder has a biological basis can be empowering for patients, as it suggests that you can work yourself or collaborate with medical professionals to restore normal functioning. I believe this represents a significant advancement in the field of neuroimaging. Although there are disorders we can not see a cause long time, we know they are related to brain changes. This represents a major step forward in our understanding and treatment of mental disorders.

**12. How do you convey your research findings to the public?**

**Ben:** I have given numerous public talks, but in China the frequency decreased significantly due to language barriers. In Germany, I often engage in such activities, collaborating with agencies that educate young people on the potential consequences of drug addiction and substance abuse. I have also served as a consultant for a film that addresses this issue, which was later screened in cinemas.

## Expert's Information

Dr Benjamin Becker is a full professor at the Department of Psychology and the State Key Laboratory of Brain and Cognitive Sciences at The University of Hong Kong. He is dedicated to researching the neural mechanisms and dynamic behavioral characteristics of emotional experiences, as well as the regulation of corresponding cognitive strategies. His adopted methods including neuroimaging, machine learning-based neural decoding and modulation, as well as other tools from human cognitive and emotional neuroscience, such as real-time neurofeedback and novel pharmacological neuromodulators. His aim is to develop innovative non-invasive treatments for mental disorders such as addiction and anxiety, and he often combines these treatments with targeted neuromodulators.

**Figure 1:**
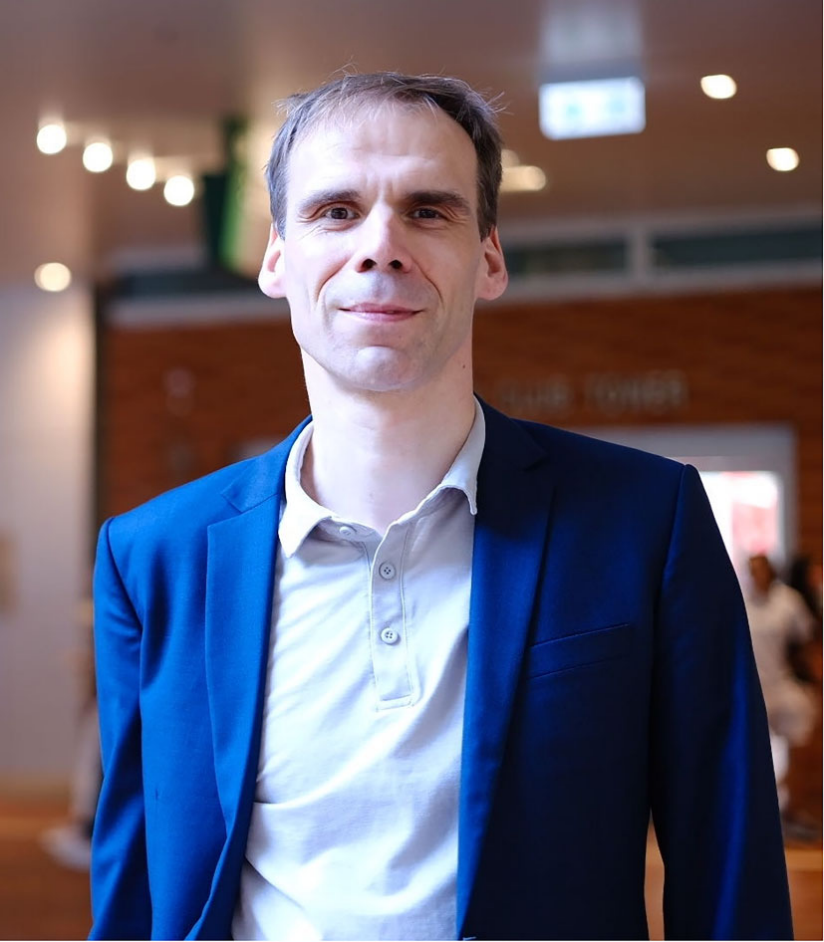


Dr Becker has published over 230 scientific papers in journals such as *Nature, Nature Human Behavior, Nature Communications, Nature Reviews in Psychology, Advanced Science, Trends in Cognitive Sciences, Proceedings of the National Academy of Sciences, USA, Molecular Psychiatry, American Journal of Psychiatry*, and *Biological Psychiatry* (>10 000 citations, h-index 54) and has been recognized as Highly Cited Researcher 2024 by Clarivate. He currently serves as Principal Editor of *Springer Psychopharmacology*, is editorial board member of *Psychotherapy and Psychosomatics* and *Psychoradiology*, and is the founding editor of *Frontiers in Social and Affective Neuroimaging*.

## Notes

Transcript edited by Lan Wang (王岚) and Shuang Luo (罗爽)

Shuang Luo (罗爽) and Lan Wang (王岚) are science and news editors at the editorial office of *Psychoradiology* in Chengdu, China.

